# Early prediction of renal parenchymal injury with serum procalcitonin

**DOI:** 10.15171/jrip.2016.23

**Published:** 2016-05-28

**Authors:** Leila Barati, Baranak Safaeian, Mahshid Mehrjerdian, Mohammad-Ali Vakili

**Affiliations:** ^1^Neonatal and Children’s Health Research Center, Golestan University of Medical Sciences, Gorgan, Iran; ^2^Health Management and Social Development Research Center, Golestan University of Medical Sciences, Gorgan, Iran

**Keywords:** Urinary tract infection, Children, Procalcitonin

## Abstract

**Introduction:** Urinary tract infection (UTI) is one of the most common bacterial infections in children that can be associated with renal parenchymal injuries and late scars. Dimercaptosuccinic acid (DMSA) renal scan is known as golden standard for detecting acute pyelonephritis (APN) that has a lot of difficulties and limitations.

**Objectives:** we designed this study the accuracy of one inflammatory marker, serum procalcitonin (PCT) to identify as an early predictor of renal injuries.

**Patients and Methods:** A prospective study was carried out in 95 patients who admitted in the hospital with the first febrile UTI. Serum PCT of all patients was measured; sensitivity, specificity, positive and negative predictive value (PPV and NPV) of this marker was analyzed compared to DMSA scan. P value <0.05 was taken as significant.

**Results:** In total, 79 females and 16 males were investigated. There are 42 cases in group 1 with normal DMSA scan and 53 patients in group two with renal parenchymal injuries in their scans. Mann-Whitney test showed a meaningful relation between the two groups regarding PCT level (*P*<0.0001). Sensitivity, specificity, PPV and NPV of PCT reported in optimum cut off were 70%, 88.1%, 88.1% and 70%, respectively. The positive likelihood ratio (PLR) of PCT test was 5.8.

**Conclusion:** In the current survey, PCT was the eligible inflammatory marker to predict renal parenchymal injuries in children with proper sensitivity, specificity, PPV and NPV that play also a pivotal role in the children aged less than 24 months, although, more studies should be undertaken to confirm.

**Implication for health policy/practice/research/medical education:**

Urinary tract infection (UTI) may present with nonspecific symptoms and its accurate diagnosis is too difficult in cases with outpatient usage of antibiotic, furthermore, delay in diagnosis and treatment of UTI in children can due to more damages in renal parenchyma. Thus finding a rapid and reliable method is a necessity. PCT level can be determined by venous blood samples that is generally available in hospitals, therefore we sought to study the value of serum procalcitonin (PCT) as an inflammatory marker in UTI.

## Introduction

Urinary tract infection (UTI) is one of the most common bacterial infections in febrile children (1). In total, 8% of females and 2% of males had history of UTI before age 7 years (2). In the first year of life, males were more involved to UTI compared to females as 2.8-5.4 to 1 times which this proportion will change to 10 more times in females after first year of life (1). Differentiation of upper and lower UTIs is difficult due to nonspecific clinical symptoms and laboratory data findings, especially in infancy. The upper UTI can be associated with renal injuries and late scars that might be resulted in irreversible effects such as hypertension and chronic renal failure (2-7). It is obvious that early diagnosis and appropriate treatment can prevent most of these complications, then, it is important to find accessible, rapid and accurate methods to identify upper UTI (4). Imaging of acute pyelonephritis (APN) was conducted using dimercaptosuccinic acid (DMSA) scan that known as golden standard for diagnosis APN and renal parenchymal injuries (2,3,6-8). The damaged locations of kidney showed a reduction of radionuclide uptake. If a DMSA scan is normal during febrile UTI, no scarring will result by that infection (9). DMSA scan has a lot of difficulties for children including high costs, time-consuming, injection and exposure to radiation. Furthermore, it has some limitations, while only 50% of children with a febrile UTI have a positive DMSA scan. Thus the benefits of alternative methods such as less damage, easier implementation, better accessibility and more accuracy will be valuable and better tolerable (3,4). Several studies have been done to investigate sensitivity and specificity of rapid diagnostic tests like serum procalcitonin (PCT) that reported controversial results (2-4,6,7,10,11). PCT is an amino acid 116 pro-peptide calcitonin without hormonal activity and its serum level is not identifiable in the usual condition or viral infections; while, PCT level increases in the bacterial infections (3,4). PCT production mechanism is still not entirely clear after inflammation. PCT in 2 hours after production of endotoxin in the blood could measurable and level of this marker usually associated with severity and mortality of disease (3-5). PCT level can be determined in early hours of injury by venous blood samples and is generally available in hospitals with limited facilities (4,5).

## Objectives

In the current survey we assayed diagnostic accuracy of PCT in renal parenchymal injuries to identify early detection renal injuries for prevention of renal scar.

## Patients and Methods

### Study population

A prospective study was performed in 95 patients who admitted with the fist febrile UTI in the Taleghani hospital (the referral hospital that affiliated to the Golestan University of Medical Sciences).

### Data collection

All patients were examined by the presumptive diagnosis of UTI, then, the urine samples obtained based on age and being toilet trained by bladder catheterization or clean mid-stream. To measure PCT, blood samples were taken from all patients. All urine samples were cultured using standard microbiological techniques. Urinary infection was diagnosed based on positive urine culture that was growth of a single bacterial pathogen more than 100000 colony-forming units (CFUs)/ml for mid-stream samples or more than 10000 CFUs/ml for catheterization. PCT level was numerically measured using electrochemiluminescence immunoassay method (Elecsys Brahms PCT kit, Roche, Germany) and considered as nanogram per milliliter. DMSA scan, was done for all patients and the renal parenchymal damages were also determined through reduced radionuclide uptake. Inclusion criteria were patients aged 1 month to 14 years with axillary temperature over 38°C and positive urine culture with one organism. Exclusion criteria were patients with a history of UTI and obstructive uropathy and kidney stones.

### Ethical issues

The research followed the tenets of the Declaration of Helsinki; all samples were participated after their parent’s satisfaction. Participation in this study was voluntary and patients were thus free to withdraw from the study at any time without having any effect on their treatment process. This study was approved by the ethic committee of Golestan University of Medical Science.

### Statistical analysis

Data were analyzed using SPSS 18 and MedCalc 11 software. The PCT normality was initially evaluated with the Kolmogorov-Smirnov and Shapiro-Wilk tests. As the hypothesis of normality was rejected, then, Mann-Whitney U test was done to measure the relation between the PCT with age and renal parenchymal injury, Chi-square test was also conducted to assess the relation of gender and renal parenchymal injury. Screening criteria were used as percentage and CI 95% including sensitivity, specificity, positive and negative predictive value (PPV, NPV). To determine the cutoff of PCT level, receiver operating characteristic (ROC) curve was used and *P* value <0.05 was taken as significant.

## Results

**Figure 1:**
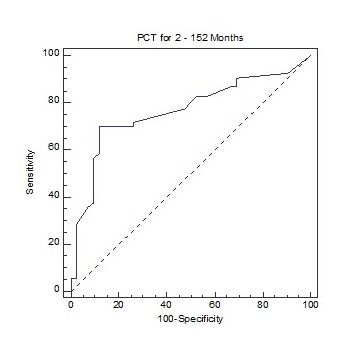
ROC curve for specificity and sensitivity of PCT measurements in total case. Area under ROC curve for PCT was 0.765 (95% CI: 0.667-0.846, *P*<0.0001).

**Figure 2:**
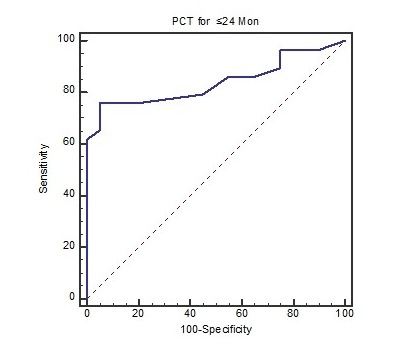
ROC curve for specificity and sensitivity of PCT measurements in children with ≤24 months. Area under ROC curve for PCT was 0.840 (95% CI: 0.707-0.929, *P*<0.0001).

In total, 95 children were studied including 79 (83.2%) females and 16 (16.8%) males. The mean age of cases was 36.2 ± 3.36 months. We divided our patients into two groups based on DMSA scan results. In the group 1, 42 patients (44.2%) had normal scan that noted the lack of renal parenchymal injury while in the group 2, 53 patients (55.8%) had renal parenchymal injuries. There was not a significant difference of gender between two groups (*P*=0.553). Mean level of PCT in group 1 was 0.1 (0.04-1.4) nanogram per milliliter and for group 2 was 0.7 (0.04-4.2). In this study, 49 (51.6%) of children were 24 months and less and 46 (48.4%) of cases were over 24 months. Chi-square test also reported that age was not significantly different between two groups (*P*=0.49). PCT level had a significant statistical difference between groups 1 and 2 (*P*<0.001; Table 1). The sensitivity, specificity, PPV, NPV and positive likelihood ratio (PLR) of PCT in children ≤24 months were significantly better than other age. The Figures 1 and 2 and Table 1 demonstrate these data. Area under ROC curve for PCT in total case was 0.765 (95% CI: 0.667-0.846, *P*<0.0001), in comparison with ≤24 months subjects that was 0.840 (95% CI: 0.707-0.929, *P*<0.0001) (Figures 1 and 2).

**Table 1:** Sensitivity, specificity, PPV, NPV, PLR of PCT in children with APN in cutoff 0.2‏ and 0.5

	**Age (month)**	**Number**	**Sensitivity (%)**	**Specificity (%)**	**PPV (%)**	**PLR**	**NPV (%)**	***P*** ** value**
PCT cut off >0.2	2-152	95	70 (55.7-81.7)	88.1 (74.4-96)	88.1 (74.4-96)	5.86	70‏ (55.7-81.7)	<0.001
	≤24	49	75.9‏ (56.5-89.7)	95 (75.1-99.9)	95.7 (78.1-99.9)	15.17	73.1 (52.2-88.4)	<0.001
	>24	46	62.5‏ (40.6-81.2)	81.8 (59.7-94.8)	79 (54.4-93.9)	3.44	66.7 (46-83.5)	0.01
PCT‏ cut off >0.5	2-152	95	53.1‏ (38.8-66.4)	90.48 (77.4-97.3)	87.5 (71-96.5)	5.5	60.1 (47.2-72.5)	<0.001
	≤24	49	57‏ (36.1-75.3)	100	100 (80.2-100)	‏-	61.6 (43-78.1)	<0.001
	>24	46	48.8‏ (27.2-68.9)	81.8 (59.7-94.8)	74.1 (46-92.4)	2.6	59 (40.1-76.2)	0.01

Abbreviations: PLR‏, positive likelihood ratio; PCT‏, procalcitonin

## Discussion

Delay in diagnosis and treatment of UTI in children can due to more damages in renal parenchymal. Since UTI may present with nonspecific symptoms and its accurate diagnosis is too difficult in cases with outpatient usage of antibiotic, finding a rapid and reliable method is a necessity. DMSA scan is gold standard to detect renal parenchymal injuries; however, it has some limitations such as high costs, not be available in all centers, time-consuming, exposure to radiation and failure to distinguish between old and new scars unless with follow-up scan (3,4,7). In the various studies, to diagnose APN, inflammatory markers have been used and compared to the DMSA scan. PCT appears in the blood in few hours after inflammation, that could be determined early renal parenchymal injury and helps physician to diagnosis and treatment of children in best time to prevent renal scar, PCT is an inflammatory marker that its sensitivity and specificity values have been investigated with controversy results, thus, we aimed to examine aforementioned findings. In this study, 55.8% of children with a first febrile UTI had renal parenchymal damages that is nearly (53%-64%) in accordance with other studies (3,4,7,13-15). Similar this study all studies showed that there were no significant difference between the two groups with damaged and normal renal parenchymal based on gender and age (3,4). In our study, the level of PCT in the two groups was statistically different which was in agreement with other studies (3,4,7,10,12,13), indicating the usefulness of this inflammatory marker to diagnose renal parenchymal damages. Sensitivity, specificity, PPV and NPV of PCT reported in the cutoff point 0.5%, 52.5%, 90.5%, 87.1% and 59.4% which had more specificity and less sensitivity compared to the Nikfar et al (4), Leroy et al (6) and Shaikh et al (13) studies, while, in Mahyar et al (2) and Güven et al (15) surveys, the sensitivity and specificity of PCT were lower compared to the current survey. In this study the cases aged 2 to 152 months were similar to other studies (2-4,12); however, no studies have been conducted to investigate the PCT in different ages. Because of the higher risk of renal injuries and scars in children younger than 24 months, in the current study PCT levels were evaluated in two groups according to age, 24 months and younger and older ones, separately. Results showed predictive value of PCT had a significant difference in the two age groups in the way that the PLR reported 15.17 in children who are 24 months and younger compared with 3.44 in older ones.

## Conclusion

This study described that the PCT as an inflammatory marker with proper sensitivity, specificity, PPV and NPV seems likely beneficial to predict renal parenchymal injury in children, which plays more vital role in children younger than 24 months, especially.

## Limitations of the study

In this project, because some of parents did not cooperate with us, we could not follow up them. Small sample of patients was another limitation of the study. We suggest multi-centric investigation on this aspect of UTI.

## Acknowledgements

The authors wish to thank Neonatal and Children’s Health Research Center, Golestan University of Medical Sciences, Gorgan, Iran, to support this study and all the colleagues and nurses who participated in the data collecting process.

## Authors’ contribution

All authors contributed to design of the research. BS, LB and MM conducted the research. MAV analyzed the data. BS and LB prepared the manuscript. All authors read, revised and approved the final manuscript.

## Conflicts of interest

The authors declare that they have no conflicting interest.

## Ethical considerations

Ethical issues (including plagiarism, data fabrication, double publication) have been completely observed by authors.

## Funding/Support

This manuscript supported financially by Neonatal and Children’s Health Research Center, Golestan University of Medical Sciences, Gorgan, Iran (Grant number #930729157).This paper is extracted from from residential thesis of Diagnostic value of procalcitonin in renal parenchymal damage in the first febrile urinary tract infection in children.
